# Breast cancer in Gaza—a public health priority in search of reliable data

**DOI:** 10.3332/ecancer.2019.964

**Published:** 2019-10-01

**Authors:** Shaymaa AlWaheidi

**Affiliations:** Cancer Epidemiology, Population and Global Health, School of Cancer and Pharmaceutical Sciences, King’s College London, London WC2R 2LS, UK

**Keywords:** breast cancer, Gaza, Palestinian authority

## Abstract

Gaza has experienced 12 years of isolation which has crippled the health system infrastructure, reduced the quality of living conditions, damaged the health of the population and reduced health service capacity and capability. This paper presents a context-setting review of what is already known about breast cancer in Gaza to identify which interventions are applicable to help prevent women there from dying unnecessarily from breast cancer. A search of the published and unpublished literature was conducted to identify potentially relevant studies on breast cancer which were either done in Gaza or elsewhere in the occupied Palestinian territory. This paper highlights the pervasive lack of basic modalities of cancer care (surgery, radiotherapy, systemic therapies and pathology/imaging) in Gaza. Poor access to breast cancer services in Gaza leaves women with only one alternative—to seek treatment outside of Gaza. However, women are sometimes forced to wait months before receiving permits to leave Gaza for treatment. Furthermore, a lack of complete and reliable data remains a major challenge for improving breast cancer services in Gaza. There is a need to develop and evaluate interventions to promote infrastructure for pathology and drug delivery, medical training and cancer registration and monitoring.

## Introduction—the political context for health and health services in Gaza

Gaza is a besieged Mediterranean coastal strip within the occupied Palestinian territory (oPt), with a complex political situation. The long-term conflict between the oPt and Israel (with major fighting in 2009, 2012 and 2014, and 2018–19 Gaza border protests) has created a fractured and a deeply unstable society [1]. Gaza has not been able to make the transition to a post-conflict context, and the continuing insecurity means there has been no opportunity for the people of Gaza to live in or to experience a recovery phase.

Gaza is home to approximately 2 million people, 70% of whom are refugees from other parts of the oPt. Unlike other refugees in the world, the Palestinians have a UN agency [UN Relief and Works Agency for Palestine Refugees in the Near East (UNRWA)] dedicated exclusively to them, and UNRWA provides them with primary health services. Cancer is the second leading cause of death in the oPt, accounting for 15% of all deaths [2], with lung cancer in men and breast cancer in women being the most common [2]. The Palestinian government’s health insurance programme covers basic cancer chemotherapy and radiotherapy for Palestinians with cancer in public hospitals in Gaza, and in those hospitals outside of Gaza to which they have been referred, while UNRWA sponsors the care of cancer patients by covering the cost of enrolling them in the government-funded health scheme [3]. Despite being demographically a young population, 32% of avoidable deaths in Gaza are attributable to non-communicable diseases such as cancer [4].

The lack of data on health performance indicators makes it difficult to comprehensively assess and compare the status of the health system in Gaza to other systems. Although substantial amounts of money are injected into the system mainly by the United Nations (e.g., UN Children’s Fund and UN Office for the Coordination of Humanitarian Affairs), the healthcare system in Gaza is fundamentally broken. The 2018 gross domestic product (GDP) was estimated to be US$14.7 billion and the GDP per capita was $3058; the public general governmental deficit is 2.8% of GDP, or about $400 million [5]. When established in 1994, the Palestinian Ministry of Health (MoH) was supported by significant international funding [6]. The healthcare system infrastructure was expanded by human resource development as well as by building clinics and furbishing wards across the three distinct regions of the Palestinian healthcare system: Gaza, the West Bank and East Jerusalem [6]. However, territorial and political fragmentation has progressively worsened despite health expenditure on medical referrals increasing [6], and the result is that health outcomes remain below the potential for the current levels of spending. At the end of July 2019, Gaza’s hospitals had run out of 516 essential medicines, representing 49% of the stock of medications included in the basic Palestinian health supply [7]. In 2015, the budget for medical referrals was GBP117 million (40% of the Palestinian Health Ministry’s budget) with 67% of the referrals funded internationally, and with borrowing (thereby incurring debt) to cover the remaining 33% [8]. The single largest specialty for the referral of patients from Gaza was cancer care, accounting for one in four patient referrals [9]. However, referral to specialist clinicians outside of Gaza is a complex process and many patients die while waiting for their approvals, which particularly affects women with breast cancer [9].

Part of the problem for women with breast cancer in Gaza is not that they have breast cancer, but more that they have to suffer this disease in Gaza while waiting for treatment outside of Gaza. Women in Gaza suffer the stigma that is associated with having the disease, and so endure the fear of embarrassment that affects their social standing in their community.

The aim of this paper is to review what is already known about breast cancer in Gaza to identify which interventions are applicable to help prevent women there dying unnecessarily from breast cancer. A search of PubMed was conducted to identify potentially relevant published studies on breast cancer which were either done in Gaza or elsewhere in the oPt. A general keyword search of breast cancer or cancer combined with Gaza or Palestinian authority were used in the search process. Reference lists of relevant literature and reports of international health and humanitarian organisations working in the oPt were scanned to identify further relevant information. Authors from Gaza were contacted to identify unpublished papers that might be relevant to this review topic.

## Women’s battle to access treatment

Ten years ago, the WHO produced an animated film called ‘Fatenah’ (https://vimeo.com/19811163) which told the story of Fatma Barghough’s battle against breast cancer. It recounted the story of the misdiagnosis of her cancer, delayed chemotherapy and the removal of both of her breasts and, because of the closure of Gaza’s borders, her inability to access the radiotherapy that she needed outside of Gaza [10]. It was partly the publicity given to her story that caused policy-makers, mainly international organisations, to put breast cancer care as a public health priority on their agendas. However, since then, very little has changed that could improve cancer treatment in Gaza. In 2017, Medical Aid for Palestinians (MAP) produced an animation (https://youtu.be/ci2BRCcRqcY) highlighting the many challenges faced by women with breast cancer in Gaza.

## Author’s experience

In January 2017, I witnessed two protests and a 1-day hunger strike outside the offices of the International Committee of the Red Cross and the Gaza-based offices of the Palestinian Civil Affairs Committee. The latter is the office that coordinates the departure of patients from Gaza with the Israeli authorities. The medication that the women required was not available, and they held up signs demanding ‘life without pain’. At that time, I was working for an International Health Organisation which provided a US Agency for International Development (USAID)-funded medical programme in Gaza that aimed to develop a programme supporting women with breast cancer. However, the organisation has effectively been shut down since September 2018 because of the cuts in US humanitarian aid to the Palestinians [33].

## Breast cancer in numbers

**Figure 1.**
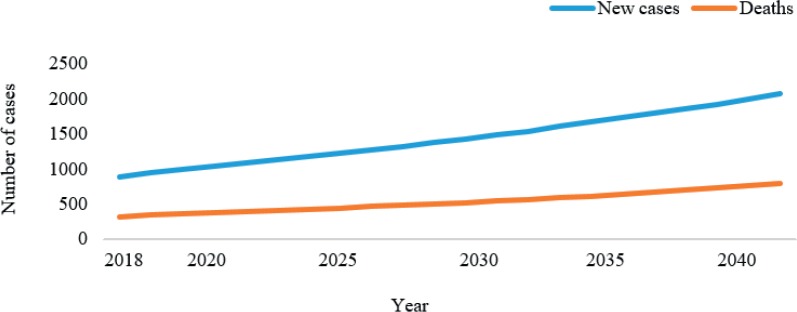
Estimated number of incident cases and deaths from 2018 to 2040, breast cancer, females, all ages, Gaza and the West Bank, cancer incidence and mortality developed by The International Agency for Research on Cancer (IARC).

As is the case in many other lower- and middle-income countries (LMICs), there is little routine data in Gaza available with which to estimate the incidence of breast cancer and trends in mortality from it. However, as far as the extant data reveals, the incidence is lower than in neighbouring countries and has been estimated to be 33 per 100,000 in 2018 [11]. However, Palestinian women’s fertility is expected to decline (the fertility rate was 4.5 in 2018) [11], and this will lead to a demographic change that will double the number of women aged 60 and over by 2050, and a more than 135% rise in breast cancer cases is expected by 2040 (Figure 1) [12]. Breast cancer mortality is also expected to increase, and the rising cancer burden will increase demands for infrastructure for pathology and drug delivery, and trained health staff accordingly. Breast cancer was the third largest cause of cancer mortality in 2018 in the oPt at 12%, after lung cancer (20%) and colon cancer (13%) [11].

Estimates vary, but the 5-year survival rate after the diagnosis of breast cancer is considerably worse than in countries with strong healthcare systems. Two research studies on cancer survival have been undertaken, with each finding a different number of women with breast cancer than those reported by the Gaza Cancer Registry. An unpublished case-note analysis by Al-Agha (2014) of 118 women diagnosed in 2007 and followed-up until 2012 yielded an estimated 5-year crude survival rate of 53% [13], while a published study by Panato *et al* [14] reported a 5-year crude survival rate of 65% for all breast cancer cases diagnosed between 2005 and 2014. This promising increase in survival may represent either an improvement in the stage at which women present to health services, their treatment, or both, or it may simply be a data artefact, as the available evidence does not suggest that either diagnosis or treatments have changed.

## Factors affecting survival among women with breast cancer in Gaza

### Social and cultural factors

Social and cultural factors impact on the stage at which women with symptoms present to the health services. Fear, embarrassment and fatalism about breast cancer are major cultural barriers in Gaza, which influence women’s decision to seek diagnosis [15–18]. In a survey by Badawi (2016) in a governmental hospital in the oPt, 54% of women with breast cancer reported delaying their visit to their doctor for more than 3 months after the onset of symptoms [15]. The majority of women interviewed said that they had noticed a change in their breast structure, but only 46% of them visited their doctors within a month of these experiences [15]. Earlier diagnosis of breast cancer cannot be achieved until women become aware of the value of early diagnosis and seek professional healthcare when they notice a change in their breasts.

### Author’s experience

I spent the summer of 2018 as a researcher at Al Rantisi Hospital in Gaza and being present in the oncology department, I had a chance to talk with many of the women who came in for treatment. I found that many women sought me out simply because I was a female health professional, and most of the women who talked to me said that they felt uncomfortable and embarrassed about approaching a male doctor for their diagnosis. They found it extremely embarrassing to talk to a male doctor about their private problems, and some even questioned the acceptability, within the context of their religious beliefs, of being examined by a male doctor.

### Resources for diagnosis and treatment

Diagnostic facilities in Gaza are scarce. Although there is no clear evidence by which to assess the quality, cost-effectiveness and accuracy of pathological services in Gaza, carrying out immunohistochemistry testing is complicated by the inadequate supply of slides and reagents, and limited human resources causes appointment delays [4]. Moreover, pathological services are conducted in a fragmented manner with no clear referral pathways among different providers [4]. Al-Shiekh [19] found that breast cancer had been missed on initial diagnostic mammography, and delays in appointments especially for biopsy were also significantly associated with delayed diagnosis. Poor survival, therefore, most probably reflects a combination of sociocultural factors and inadequate and delayed diagnosis.

To a lesser degree, survival rates will also be reduced by the lack of basic chemotherapy which is only intermittently available. More than half of basic chemotherapy drugs were at less than a month’s supply in 2018, and radiotherapy, a major determinant of survival, is completely unavailable.

The waiting time for breast cancer surgery at the governmental Al-Shifa Hospital now stands at up to 9 months [4], a delay that has a major impact on overall survival for early-stage breast cancers. In Gaza, up to 80% of women requiring surgery receive total mastectomies for breast cancer, which has been mainly justified on the lack of radiotherapy [20, 21], despite the almost global change to lumpectomy and wide local excision for the majority of cases as the standard of care. Furthermore, there are no surgeons specialising in breast cancer, especially oncoplastic reconstruction, with expert surgical care being provided intermittently by visiting volunteers, mainly through MAP which also facilitates knowledge-sharing between British and Palestinian oncologists and radiologists through fellowships in the UK [22].

### Referral for radiotherapy

Cancer patients are usually referred to Augusta Victoria Hospital in Jerusalem for radiotherapy and to Israeli hospitals for complex imaging such as positron emission tomography -computed tomography (PET-CT) [2]. However, referral is a chaotic process that includes: 1) perceptions of discrimination and preferential referral approvals to more patients from the West Bank over patients from Gaza; 2) delays in appointments and 3) waiting for many months to receive permits from Israel to leave Gaza through the Erez Checkpoint, with no information about necessary follow-up action being available [4, 23]. In addition, depending on the political situation, the border crossing-points are closed to patients with permits at very short notice thereby ensuring that any posttreatment complications cannot be managed in a timely fashion. On average, there are about 2,000 permit requests made per month, and according to the WHO 2018 report, the number of referrals denied by the Israeli authorities in 2018 rose by 180% compared to 2017. In July 2019, 68% of patients applying for a medical exit permit received one in time for their medical appointment, 12% were denied and 20% were delayed access to care, receiving no response to their application by the date of their treatment appointment [24]. Physicians for Human Rights Israel mentions that some Israeli medical exit permit policies affect women twice as often as men, and that in 2017–2018, 129 women from Gaza contacted them after their permit to receive needed treatment was delayed or denied [25]. The effects of Israel’s permit policy can be seen in cases such as that of Khadijah, a 32-year-old mother of four suffering from breast cancer [26]. She requested a medical exit permit for scans in East Jerusalem in January 2018 and was rejected. In July, Khadijah reapplied for a medical permit for surgery and again was rejected. She then decided to change her treatment destination to Egypt, and 7 months after diagnosis, Khadijah had surgery in Egypt [26].

Even after women manage to receive a medical permit, their treatment places a severe financial hardship on the Palestinian government. When a patient is referred to a nongovernmental hospital for treatment, the average breast cancer case costs the MoH up to £70,000 [4], and this is double the cost that the MoH would have spent if the patient had been treated in its own hospitals. There may also a substantial social and psychological burden on women themselves and their families. However, this is not being addressed in current research. Women with breast cancer are sometimes forced to travel alone when Israel refuses to approve exit permits for relatives to accompany them during their treatment periods. This could affect their mental health and treatment outcomes while being treated away from their children and families for many weeks at a time.

## Health research capacity

Before 2010, breast cancer epidemiology was documented in reports of the Palestinian MoH and in the reports of international organisations. Most research conducted in Gaza was cross-sectional, and it studied risk factors associated with breast cancer and factors affecting the stage of diagnosis (such as women’s awareness level of early detection methods, and mainly of *ad hoc* mammographic screening). The research conducted did not fully address the concerns that women have expressed regarding treatment availability and accessibility in Gaza. As in many other LMICs, Gaza still lacks sufficient health research capacity to build a local evidence-base with which to inform policy and to improve population health. Many studies conducted on breast cancer are unpublished master’s theses which could mean that they are less accessible to the general public or to policy-makers, and thus they do not influence care provision. Another problem is that the evidence on breast cancer in Gaza, as is the case with most countries in the Middle East, is not translated into the language that the women themselves speak. This means that they cannot benefit from the research that is conducted in their own countries.

One of the many challenges in undertaking research of good quality and of achieving service improvement for breast cancer in Gaza is the lack of reliable data, and the capacity to make use of such data by researchers. While a very considerable amount of funding has been allocated to establish electronic cancer records, it remains the case that to get information about breast cancer from hospital notes, from death certificates, and from referral reports, requires manual abstraction and trained staff.

## A way forward

- Support cancer registration.
- Strengthen human resources for breast healthcare.
- Promote women’s awareness of breast cancer and encourage early presentation.
- Support infrastructure for pathology services and regular drug delivery.
- Improve research capacity and oncology-related economic assessments.
- Invest in partnerships and collaborative cancer policies.

### Cancer registration

First and perhaps foremost is the need for a population-based cancer registry for Gaza that will allow policy-makers to properly plan cancer care services based on empirical evidence of burden. There have been many efforts to establish electronic cancer records which can be highly useful for building service quality, but the assistance offered has not been sufficient, and the reasons for this have not been investigated. Although finance is an important factor to purchase technology, electronic health information systems can only be built efficiently and effectively when there are preexisting well-maintained paper records of diagnosed cases based on high-quality diagnostics (pathology and imaging). There must then also be health and administrative staff who have the time, sufficient training and financial resources (such as office space, computers and electricity) that are needed for the implementation of new policies; support which is still not available in Gaza [27]. In their systematic review, Akhlaq *et al* [28] concluded that many LMICs have overcome these barriers through clear policy direction that has enabled them to hire and train extra staff to help overburdened professionals. In addition, there has been capacity building such as the provision of follow-up of training, and coaching to gradually change attitudes towards the use of electronic health information systems.

### Research capacity building

To increase the likelihood of future breast cancer policies being effective, there is also a need to prioritise and build research and quality improvement expertise so that efforts can focus on questions with the greatest clinical relevance to breast cancer in Gaza. Such an approach would ensure that research findings can be used to inform local breast cancer care practice. For example, an audit of current internationally accepted treatment of women with breast cancer is needed to identify deficiencies and to investigate adherence to treatment and surgical guidelines by surgeons and oncologists. Further assessment of the quality, cost-effectiveness and accuracy of pathological services in Gaza is still required to inform the most appropriate management of treatment for women with breast cancer. This might help to inform an economic assessment of actual need of treatment facilities which could influence how the government-funded treatment budget could be spent in the most cost-effective manner.

### Investment in public awareness and resources for breast cancer care

Given the major barriers to research in Gaza, there is a need to learn from past experiences in other resource-limited settings [29–31]. For example, taking stock of past experiences in such settings regarding breast health awareness interventions could help identify evidence-based and locally relevant interventions to educate women in Gaza about breast symptoms and encourage early presentation to the health services [32]. Women will then be empowered to make better health choices about presentation and treatment and to advocate for higher quality services.

The maintenance of regular drug supplies would help reduce the number of patients applying for exit permits to exit Gaza to access needed chemotherapy. It will also subsequently reduce delays in care and the financial burdens that are being placed on the MoH. Because of the political and economic contexts, some of the challenges (e.g., the unavailability of radiotherapy, and patient permit delays and rejections) will be unsolvable without the cooperation and encouragement of the international community to affect such change. An investment in cancer control through partnerships between Gaza and high-income countries should be encouraged to provide training courses in radiology, radiography, surgery and other aspects of cancer care. However, vigilance is required to ensure that the goals of such collaborations are aligned with the needs of the women we seek to help.

## Conclusion

As elsewhere in the world, access to healthcare in Gaza is important, but it is also complex because it is affected by conflicts in the area, restrictions on travel for treatment, and lack of expertise and resources for pathology and treatments. Although there has been descriptive research describing the poor survival of women diagnosed with breast cancer in Gaza, the factors that contribute to this state of affairs have not been systematically investigated despite the MoH’s attempts to support cancer treatment in Gaza. Alongside breast cancer registration and monitoring, collaborative research and medical training, resources for prompt diagnosis and regular drug supply are needed now more than ever in the face of demographic changes in the oPt and the subsequent increase in cancer incidence and mortality.

## Conflicts of interest

The authors declare that they have no conflicts of interest.

## Funding declaration

This publication is funded through the UK Research and Innovation GCRF Research for Health in Conflict (R4HC-MENA); developing capability, partnerships and research in the Middle and Near East (MENA) ES/P010962/1.
